# Burnout in primary healthcare physicians and nurses in Turkey during COVID-19 pandemic

**DOI:** 10.1017/S146342362200069X

**Published:** 2023-01-09

**Authors:** Ata Arda Ayaslıer, Beyza Albayrak, Esra Çelik, Özgür Özdemir, Özlem Özgür, Emrah Kırımlı, İlker Kayı, Sibel Sakarya

**Affiliations:** 1 School of Medicine, Medical Student, Koç University, Istanbul, Turkey; 2 Ümraniye Family Health Center, Family Physician, Istanbul, Turkey; 3 Department of Public Health, School of Medicine, Koç University, Istanbul, Turkey

**Keywords:** family physicians, Maslach burnout inventory, primary healthcare nurses, Turkey

## Abstract

**Background:**

Due to additional responsibilities and uncertainties during the COVID-19 pandemic, primary healthcare (PHC) workers are at increased risk of burnout.

**Aim:**

To determine and compare the burnout levels and related factors in PHC nurses and family physicians (FPs) during the COVID-19 pandemic.

**Methods:**

An online survey was delivered to PHC workers. Non-random sampling method was used. To evaluate burnout, the Maslach Burnout Inventory was used, which investigates burnout in three categories: emotional exhaustion (EE), depersonalization (DP) and reduced personal accomplishment (PA). Multivariate linear regression was used to analyze factors associated with burnout for FPs and nurses separately.

**Findings:**

Among the participants, 55.7% were nurses, the mean age was 42.34. FPs and nurses experienced similar levels of burnout in terms of EE. Family physicians had higher levels of low PA and DP. Based on the results of the multivariate analysis, while higher EE levels were significantly associated with unequal distribution of workload and communication problems within the Family Health Center for physicians, the unequal distribution of PPE, lack of appreciation by patients or colleagues and restrictions on work-related rights were relevant factors for nurses. Lack of appreciation and restrictions of the rights were associated with increased DP scores in both groups. Unequal distribution of workload was also associated with reduced PA among FPs.

**Conclusion:**

PHC physicians and nurses are affected by burnout in different ways under the conditions of the COVID-19 pandemic based on gender, socioeconomic status and working conditions. To protect the mental health of PHC workers in the next public health emergency, clarification in the organization of services, empowering PHC workers in emergency risk communication and provision of timely, adequate and free PPE is essential. It is also crucial to ensure the rights of health workers through macro policy changes especially during emergencies.

## Introduction

During the COVID-19 pandemic, healthcare workers have been experiencing a considerable psychological strain (Matsuo *et al.*, 2020). Since adverse psychological reactions in healthcare workers were reported in the 2003 SARS outbreak (Lai *et al.*, 2020), the psychological condition of Health Care Workers (HCW) have been an important issue due to concerns about health system capacity. Burnout, which is defined by Christina Maslach as a ‘psychological syndrome of emotional exhaustion (EE), depersonalization (DP) and reduced personal accomplishment (PA)’ (Maslach, 1993), is one of the major risks for healthcare workers. An example is the study of Matsuo *et al.* (2020) during the COVID-19 pandemic, which showed that more than 40% of nurses and more than 30% of radiological technicians and pharmacists met the criteria for burnout according to Maslach Burnout Inventory (MBI). Burnout in HCWs can be linked to a variety of reasons such as excessive workload and interpersonal conflict in the workspace and can lead to serious outcomes including medical errors/accidents (Kitaoka and Masuda, 2013).

As the World Health Organization (WHO) indicates, primary health care (PHC) plays an important role in the COVID-19 response through early diagnosis and reducing the demand for hospital services (WHO, 2020). Due to the additional responsibilities and consequent unfavorable working conditions, PHC workers have an increased risk for burnout during COVID-19 pandemic. According to a study conducted to identify the immediate support needs of Australian primary care nurses during the COVID-19 pandemic, there were 7 categories identified as protective equipment, communication, self-care, valuing nurses, industrial issues, workplace factors and financing (Halcomb *et al.*, 2020). Such factors in many countries pose a risk for PHC workers for physical and mental strain, and increased levels of burnout. For example, a study conducted in general physicians in Italy during COVID-19 pandemic by Di Monte *et al.* (2020) has shown that 46.1% of the participants were in the high-burnout group for EE, 42.2% reduced PA and 17.6% for DP.

Family Health Centers (FHC) in Turkey consist of one or more core teams, each of which are comprised of a family physician (FP) and a nurse or midwife, the responsibility for the execution of the FHC belongs to the FP. The Ministry of Health (MoH) employs FPs as contracted personnel and pay per capita based on the number of registered people (Akman *et al.*, 2017). Like other countries, the workload due to the pandemic control measures and psychological stress of healthcare workers working in PHC increased during the COVID-19 response efforts in Turkey. In a study conducted in Turkey, it was shown that both job strain and anxiety levels increased significantly during the pandemic process (Taş *et al.*, 2021). According to a survey conducted by the FPs Working Group of Turkish Medical Association (TMA) with 1270 FPs in November 2020, the rate of those who said they have never felt burnout was only 2%, while the rate of FPs who were exhausted from time to time, most of the time, and completely was 32%, 66% and 18%, respectively (Family Physicians Working Group of TMA, 2020). Another study evaluating the level of burnout in healthcare workers using MBI during the COVID-19 pandemic also showed that women, younger people, primary care workers and frontline workers were at higher risk of burnout (Kılıç *et al.*, 2021).

Regarding the role of the primary care in the management of the pandemic in Turkey, there was a divide between hospital based care and primary care (Kayı, 2020). Case management guidelines were prepared for the hospital setting leaving the primary care out of the picture. Since COVID-19 testing was only performed in hospital setting, patients admitting to the FHC with COVID-19-like symptoms had to be referred to hospitals by their FPs; however, due to fear of infection many patients have chosen to seek medical care at the primary care level and avoided going to the hospitals. Due to the generally inadequate physical conditions of FHCs along with shortages in personal protective equipment, overcrowding of the centers increased the risk of transmission and created difficulties to manage the operations at primary care. Division of labor at the FHCs was another issue. For example, one task assigned to FPs was following-up isolated and quarantined cases at home by making periodic phone calls. However, there was no standard operational procedures, and at some centers, FPs have delegated this task to the nurses they work with. If not for the tasks assigned by the FPs, officially there was no duty primary care nurses were expected to fulfill in terms of COVID-19 prevention or case management until the COVID-19 vaccination campaign started. Most of the time nurses tried to continue their pregnancy follow-ups and childhood immunizations by creating a safe space within the FHC.

The aim of our study is to determine and compare the burnout levels and related factors in PHC nurses and physicians during the COVID-19 pandemic in Turkey.

## Methods

This is a cross-sectional study, in which primary care physicians and nurses in Turkey were the target population. There are approximately 22,781 PHC physicians and 21,302 PHC nurses/midwives in Turkey (Turkish MoH, 2019). In this article, the term ‘nurses’ is used for the nurses and midwives participating in the study.

We used a non-probabilistic sampling technique to recruit PHC workers in Turkey. Data were collected via an online survey, which was pilot tested in one of the FHCs in Istanbul with 6 participants. After the corrections on the survey, final version was distributed by three contact persons (two PHC nurses and one FPs) in their professional Whatsapp groups with members from various parts of Turkey to reach out to PHC workers in Turkey. The survey was open for four weeks between February 2021 and March 2021 with weekly reminders. Informed consent was obtained from all subjects at the beginning of the online survey.

At the time of the data collection, Turkey was experiencing the third peak of the COVID-19 pandemic, and as a priority group, healthcare workers were vaccinated with the full dose of inactivated virus vaccine available in Turkey (Ritchie *et al.*, 2020).

The main dependent and independent variables were burnout level of the participants and their professions, respectively. Survey questions included sociodemographic information (age, gender, marital status, region of residence), MBI, perceptions about the seriousness of the disease, experience of contracting the disease or staying in quarantine, factors complicated the working conditions during the COVID-19 pandemic environment, concerns regarding the pandemic, and suggestions for improvement. Questions about the factors that complicated working conditions were largely prepared based on the survey conducted by the FPs Working Group of TMA among FPs during the pandemic (Family Physicians Working Group of TMA, 2020). In addition, the needs expressed by healthcare professionals on social media during the pandemic were also used as a list of items for participants to choose whichever applies.

MBI is a Likert type scale with 22 items, and it has been validated into Turkish by Ergin (Ergin, 1992). MBI is composed of three dimensions: EE (9 items), DP (8 items), and reduced PA (5 items) (Maslach and Jackson, 1981). Choices in MBI from never to always were assigned values from 0 to 4, respectively. To evaluate reduced PA, the calculations were done by inverse scoring of the options (eg ‘Always’ = 0).

Apart from the Maslach Burnout Scale, we asked the participants their opinion on their burnout level with five options: ‘Which of the options below do you think best explains your level of burnout?’ (‘I don’t feel burnout, I enjoy my job’, ‘I feel stressed and low on energy from time to time, but I do not feel exhausted’, ‘I feel exhausted and show one or more signs of burnout’, ‘The burnout symptoms I show never improve. I often think about unrest at work’ and ‘I feel completely exhausted, and I don’t know if I can go on like this’).

For data analysis, SPSS 26 for Windows was used. For the MBI subscale score the median and interquartile range were used, since the distribution was non-normal. Mann–Whitney U test and Kruskal–Wallis test were used for statistical analysis. Multivariate linear regression analyses were performed for FPs and nurses separately to analyze the factors associated with the three subscales of MBI namely EE, DP and reduced PA. Age, living with a person from risk groups for COVID-19, and factors complicating working conditions during the COVID-19 pandemic were included in the model as independent variables.

## Results

We received 745 initial responses from a total of seven regions in Turkey and 64 of 81 provinces. After 163 people were excluded due to missing responses in MBI survey, 582 people were included in the analysis. Among these, 44.3% were FPs (*n* = 258) and 55.7% were nurses (*n* = 324). The proportion of women among nurses was 97.8%, while among physicians was 43.6%. The mean age of the FPs was greater than the nurses (47.3 vs 38.3; *p* < 0.05). Average years of service were around 10 years in both professions; FPs, on the other hand, were slightly older in the profession (*p* < 0.05). Almost 60% of the nurses were from the Marmara region, whereas physicians participated mostly from Marmara (35.1%) and Aegean regions (29.6%) (*p* < 0.05). Most of the respondents were married; 87.5% of the FPs and 80.7% of the nurses had children (*p* < 0.05) (Table 1).

**Table 1.** Demographic characteristics of respondents

	**Family physician (** ***n*** **= 258)**	**Nurse (** ***n*** **= 324)**
**Gender** ^*^ (*n*,%)		
Female	112 (43.6)	318 (97.8)
Male	145 (56.4)	3 (0.9)
Self-Declared	–	4 (1.2)
**Age** ^*^ **(**Mean ± SD)	47.3 (7.6)	38.3 (6.7)
**Years in Service** ^*^ (Mean ± SD)	10.5 (4.9)	9.0 (4.9)
**Marital Status** (*n*, %)		
Married	217 (84.4)	259 (80.7)
Single	40 (15.6)	62 (19.3)
**Parental Status** ^*^ (*n*, %)		
Yes	224 (87.5)	263 (81.2)
No	32 (12.5)	61 (18.8)
**Geographic Distribution** ^*^ (*n*, %)		
Marmara	90 (35.0)	193 (59.8)
Central Anatolia	24 (9.3)	31 (9.6)
Aegean	76 (29.6)	29 (9.0)
Mediterranean	39 (15.2)	28 (8.7)
Black Sea	18 (7.0)	23 (7.1)
Eastern Anatolia	7 (2.7)	8 (2.5)
Southeastern Anatolia	3 (1.2)	11 (3.4)

* *p* < 0.05.

COVID-19 diagnosis was reported by FPs and nurses as 17.1% vs 18.2% and quarantine rates as 17.5% vs 22.2%, respectively. Majority of FPs (76.5%) and nurses (78.4%) perceived COVID-19 disease as a severe disease. The proportion of nurses living with people from the COVID-19 risk group was higher than FPs (50.3% and 42.4%, respectively; *p* < 0.05). The proportion of nurses who considered resigning during the COVID-19 pandemic was also higher than FPs (48.3% and 38.1% respectively; *p* < 0.05) (Table 2).

**Table 2.** Perceptions and experiences of the participants about the COVID-19 disease

	Family Physician (*n* = 258)	Nurse (*n* = 324)
**Perception about nature of** COVID**-19 disease (** ***n*** **,%)**		
Mild	5 (2.0)	7 (2.2)
Moderate	55 (21.6)	61 (19.4)
Severe	195 (76.5)	247 (78.4)
I have been diagnosed with COVID-19 disease **(** ***n*** **,%)**	44 (17.1)	59 (18.2)
I’ve been quarantined **(** ***n*** **,%)**	45 (17.5)	72 (22.2)
Someone in my family or close circle has been diagnosed with COVID positive **(** ***n*** **,%)**	192 (74.4)	243 (75.0)
Among the people I have lived with, there are those in the risk group for COVID-19 disease **(** ***n*** **,%)** ^*^	**109 (42.4)**	**163 (50.3)**
I thought about retiring during the COVID-19 pandemic^*^	**98 (38.1)**	**155 (48.3)**

* *p* < 0.05.

The analysis of PHC workers’ opinion about their own burnout showed that 3.1% of FPs and 1.9% of nurses reported that they did not feel burnout. On the other hand, 13.8% of the nurses and 4.7% of the FPs reported that they felt completely exhausted (*p* < 0.05) (Table 3).

**Table 3.** Self-reported health status and burnout subscales among family physicians and nurses

Which of the following options do you think best explains your burnout level? (%)	Family physician	Nurse	*p* ^*^
I don’t feel burnt out, I enjoy my job.	3.1	1.9	**0.005**
I feel stressed and low on energy from time to time, but I do not feel exhausted.	37.7	32.1	
I feel exhausted and show one or more signs of burnout (physical and emotional fatigue).	44.4	41.7	
The burnout symptoms I show never improve. I often think about unrest at work.	10.1	10.5	
I feel completely exhausted, and I don’t know if I can go on like this.	4.7	13.9	
**Burnout subscales (MBI) (median, IQR)**	Family physician	Nurse	*p* **
EE (9 questions, max score: 36)	20.0 (8.5)	21.0 (8.5)	0.364
DP (5 questions, max score: 20)	7.0 (5.0)	6.0 (5.0)	**<0.001**
Reduced PA (8 questions, max score: 32)	12.0 (5.0)	11.0 (5.0)	**0.042**

* chi-square test

** Mann–Whitney *U* test.

Regarding the MBI scores, FPs and nurses did not differ in EE (*p* > 0.05); however, FPs were experiencing significantly higher degrees of DP and reduced PA compared to nurses (Table 3).

In univariate analysis, a weak and negative correlation was found between age and EE only among FPs (*r* = -0.15; *p* < 0.05). Living with a person from risk groups for COVID-19, such as chronic illness or old age, was associated with higher EE levels only among nurses (*p* < 0.05) There was no statistically significant difference between regions and burnout in both groups (data not shown).

**Figure 1.**
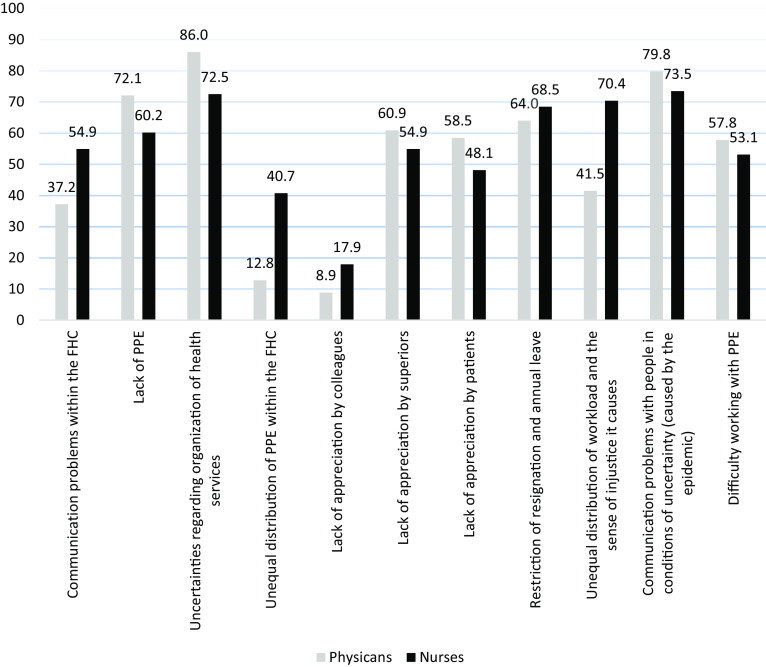
Factors complicated the working conditions during the COVID-19 pandemic environment (%)

Figure 1 shows the factors reported to complicate working conditions in the COVID-19 pandemic environment. FPs and nurses differed in all factors (*p* < 0.05) with the exceptions of *‘the difficulty of working with PPE’, ‘lack of appreciation by superiors’* and *‘resignation and annual leave restriction’* (*p* > 0.05) (Figure 1).

Multivariate linear regression analysis results are presented in Table 4. For FPs, ‘*unequal distribution of workload and the sense of injustice it causes*’ was found to be significantly associated with higher burnout levels in all three dimensions of the MBI scale. ‘C*ommunication problems within the FHC’* was the other factor associated with higher EE levels. On the other hand*, ‘lack of appreciation by patients or superiors’* and *‘restriction of resignation and annual leave’* were the factors associated with higher level of DP.

**Table 4.** Factors associated with MBI subscales among FPs and nurses (multivariate linear regression analysis results)

Variables	Family physicians						Nurses					
	EE		DP		PA		EE		DP		PA	
	β	*p*	β	*p*	β	*p*	β	*p*	β	*p*	β	*p*
Unequal distribution of workload and the sense of injustice it causes	3.0	<0.001	1.6	0.002	1.2	0.023						
Communication problems within the FHC	2.0	0.02										
Unequal distribution of PPE within the FHC							1.9	0.009				
Lack of appreciation by patients			1.2	0.020			1.9	0.006	1.2	0.004		
Lack of appreciation by superiors			1.0	0.04								
Lack of appreciation by colleagues							2.3	.008	2.3	<0.001		
Restriction of resignation and annual leave			1.3	0.008			2.0	0.006				

For nurses, ‘*unequal distribution of PPE within the FHC’,* ‘*lack of appreciation by colleagues or patients’ and ‘restriction of resignation and annual leave’* were the factors associated with higher level of EE. ‘*Lack of appreciation by colleagues or patients’* was also associated with higher DP levels. There were no factors associated with reduced PA (Table 4).

Age and living with a person from risk groups for COVID-19 were not associated with burnout in both groups.

**Figure 2.**
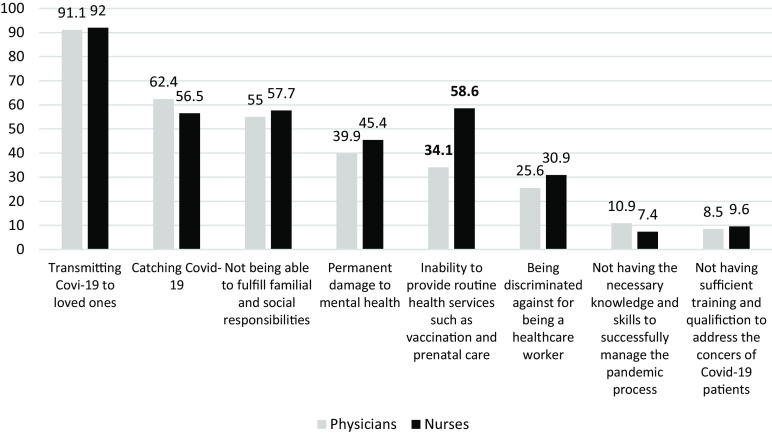
Physicians and nurses’ concerns about the COVID-19 pandemic (%)

We asked the participants on their concerns regarding the pandemic and the subsequent conditions. The primary concern for both physicians and nurses was *‘transmitting COVID-19 to loved ones’ (91.1% vs 92%, respectively). ‘Concern about inability to provide routine health services’* was 34.1% among FPs and 58.6% among nurses (*p* < 0.05). Apart from this factor, the responses of physicians and nurses were similar (*p* > 0.05) (Figure 2).

**Figure 3.**
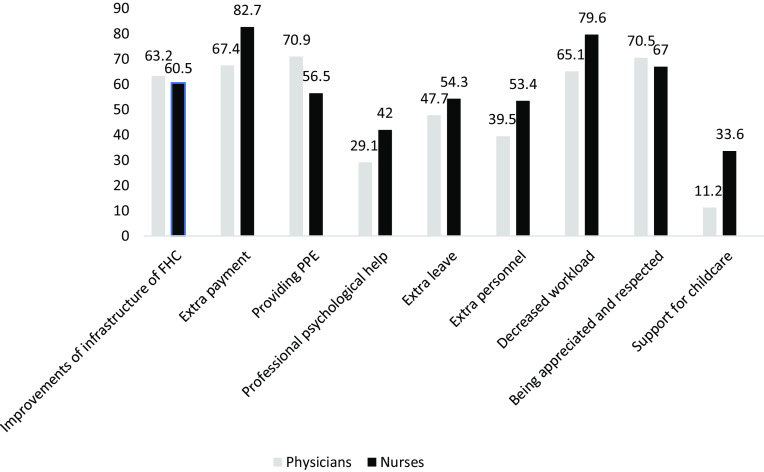
Suggestions for improvements during COVID-19 pandemic

We also asked the participants to choose three among ten choices that would help them to improve their working/life conditions. Among the FPs the three most common chosen options were *‘PPEs being provided’* (70.9%), *‘being appreciated and respected’* (70.5%) and *‘receiving extra payment’* (67.4%). The top three choices of the nurses were: *‘receiving extra payment’* (82.7%), *‘decreased workload*’ (%79.6) and *‘being appreciated and respected’* (67.0%) (Figure 3).

## Discussion

In this study, which was conducted approximately in the first year of the COVID-19 pandemic, we examined the factors associated with burnout levels among primary care physicians and nurses/midwives. The results of the study indicate that while there are some common factors related to burnout experienced among PHC workers during the COVID-19 pandemic, there are also important differences between the two groups.

FPs and nurses were similar in their perceptions of the severity of the COVID-19 disease and their experiences of contracting the disease and/or staying in quarantine.

According to the primary care workers’ opinion on their burnout level, rate of ‘feeling completely exhausted’ in nurses was 3 times higher than that of physicians. Similarly, the rate of those considering resignation among nurses was 1.3 times higher than physicians. High burnout levels of nurses have been shown in previous studies both during and before the COVID-19 pandemic that showed associations between burnout and social, economic and gender-based inequalities in the society (Cam and Yıldırım, 2010; Pappa *et al.*, 2020; Abraham *et al.*, 2021; Uz *et al.*, 2022). One of these studies from Turkey concluded that female healthcare workers, nurses and medical secretaries experienced higher levels of mental health symptoms than male healthcare workers and physicians (Uz *et al.*, 2022). Also, Tarhan *et al.* observed that the burnout among physicians during the COVID-19 outbreak was higher in women (Tarhan *et al.*, 2021). Similarly, in a countrywide population-based study from Turkey, it was found that females report lower mental health status than males (Kose, 2020). As most of our nurse participants were female (97.8%), our findings reflect the already existing gender difference in the mental health status of individuals in the population (Albert, 2015; Kose, 2020).

In our study, FPs and nurses experienced similar levels of burnout in terms of EE. However, DP and reduced PA were significantly higher among physicians. In a similar study conducted among PHC workers before the COVID-19 pandemic, it was found that the EE and DP scores of FPs were significantly higher, and the sense of PA scores was considerably lower compared to family health workers (nurses and midwives) (Emre *et al.*, 2020). There are other studies done prior to the pandemic that show higher levels of burnout for physicians than for other professionals (Elbarazi *et al.*, 2017; Zarei *et al.*, 2019).

The EE scores showing no difference between FPs and nurses – unlike previous studies – may be due to the fact that our study was conducted during the COVID-19 period which had exacerbated the gender-based inequalities. In our study while majority of the nurses were females, only half of the physicians were females. For example, childcare, which mainly falls on the shoulders of women, has been a major problem in Turkey due to the prolonged closure of schools and nurseries (Özer *et al.*, 2022). There are also gender differences in terms of mental well-being in favor of men as mentioned earlier. Therefore, women’s deteriorating EE levels might be associated preexisting emotional problems. Apart from gender difference, there is also an income gap between nurses and FPs, which might affect the management of everyday life during the pandemic (ie child or elderly care) in a negative way for nurses.

In our study, while there was no significant difference in EE levels between physicians and nurses, determinants associated with higher EE lives were different in the two groups. Unequal distribution of PPE within the FHC, lack of appreciation by colleagues or patients and restriction of resignation and annual leave were associated with higher levels of EE only among nurses. Although both FPs and nurses stated ‘*lack of PPE*’ in FHC as a problem with high proportions, nurses have complained about ‘*unequal distribution of PPEs within the FHC*’ more than FPs (40.7% vs 12.8%). The reason of this unequal distribution might be related to the fiscal management of FHCs in Turkey, where MoH allocates a budget for FHCs paid only to FPs for disposable materials end everyday operational expenditures (Öcek *et al.*, 2014). Therefore, purchasing PPE and their distribution in FHC during the pandemic was considered as responsibility of FPs. Such difference in fiscal control in FHCs makes the FPs relatively advantageous in access to PPEs. Hence, such conditions perceived by nurses might have affected their EE levels more compared to FPs.

On the other hand, for FPs, there were two distinct issues that were found to be associated with EE in the workplace: communication problems within the FHC and unequal distribution of workload and the sense of injustice it causes. Communication problems reported by physicians may refer to communication problems both within the PHC team and with patients. In both cases, it is known that communication problems affect job satisfaction and burnout level. In a study conducted in Turkey comparing burnout levels and communication skills in primary care staff; while it was found that the competency levels in communication skills were inversely associated with EE, DP and PA levels, it was also found that the female physicians and the family health workers (nurses and midwives) had better communication skills than male physicians (Emre *et al.*, 2020). The association between reported communication problems and higher EE levels among FPs in our study might be due to the higher male presence in the physician group compared to nurses.

According to MBI results, FPs had higher burnout levels in terms of DP and reduced PA subscales. It has been widely acknowledged that pandemic created a process of uncertainty as the SARS-CoV-2 was a novel pathogen followed by is variants, which brought continuous need for information. Therefore, the management of the pandemic has been difficult in terms of decision-making. In the PHC context, the managerial responsibility of physicians and being responsible for many decisions in a time of uncertainty may be related to significant difference in DP levels between FPs and nurses (Di Trani *et al.*, 2021). Since the onset of the COVID-19 pandemic, MoH of Turkey has chosen to manage the system almost entirely with a hospital-oriented approach (Kayı, 2020), thus creating a certain uncertainty about how the primary care services will be carried out. Most of the uncertainties in Turkey was due to lack of well-structured short- and long-term action plans regarding the role of PHC facilities and the organization of routine health services specially at the onset of the pandemic. To eliminate this deficiency, TMA Family Medicine Branch has prepared a guide on the organization of PHC services in the pandemic and made it available to FPs (TMA-FM, Guide for COVID-19 Pandemic 2020); however without the recognition of MoH, it was mostly up to the FPs to decide how to operate. In a study conducted in Bulgaria with 146 healthcare professionals, manageability dimension of sense of coherence was shown to be significantly associated with all dimensions of burnout including DP (Stoyanova and Stoyanov, 2021). Our findings indicate that the issue of uncertainties regarding organization of healthcare services was reported as the most common problem by the FPs and the second most common problem by the nurses, supporting the idea that managerial responsibility falling on the FPs might be an important contributing factor for higher DP levels in FPs.

The restriction of the resignation and annual leave during the pandemic period was associated with higher burnout scores in terms of EE and DP levels among both FPs and nurses alike. According to MoH data, 2,412 doctors resigned between March 2020 and September 2020 in Turkey, of which 1410 are general practitioners (Duvar English, 2021). Thereupon, with the circular published by the MoH in October 2020, restrictions were imposed on the resignation, retirement and leave rights of health workers during the pandemic process (TNA, 2020). Such legislations by the MoH can be considered as a limitation on health professionals’ sense of control over their job, which might be the reason behind the association of this restriction with higher EE and DP levels among both FPs and nurses.

As seen in Table 4, ‘not being appreciated by the patients or superiors or colleagues’ were factors associated with high DP among both physicians and nurses. Similar results were reported in another study from Turkey during the COVID-19 pandemic (Tarhan *et al.*, 2021). Accordingly, physicians who are not appreciated by their superiors have a higher burnout level and they clearly state that they are exhausted because they are not rewarded for their work, either financially or morally. It should be noted that the ‘lack of appreciation’ by superiors and patients voiced by FPs and nurses in our study was on the agenda of healthcare professionals before the COVID-19 pandemic. Health workers have been struggling for their rights through professional organizations due to the increasing violence in healthcare environment, poor economic and working conditions (TMA, 2007, 2013, 2017; Ekmek ve Gül, 2020). It would not be wrong to assume that the COVID-19 pandemic has deepened the existing problems with the increase in workload and uncertainties.

Different from EE and DP dimensions of burnout, reduced PA was associated with a limited number of the work-related factors. The association of reduced PA was significant with unequal distribution of workload and the sense of injustice it causes only in FPs. PA is related to people’s feelings of competence in their jobs and their motivation for success (Maslach and Jackson, 1981). It can be said that the pressure of high uncertainty and scarcity of resources created by the COVID-19 pandemic (Koffman et al., 2020; Di Tirani *et al.*, 2021) makes it difficult for physicians to fulfill their work-related responsibilities and therefore reduces self-confidence and motivation for success.

This study is subject to several limitations. As the recruitment was based on non-random sampling, the results of our study might fall short of representing our target population due to the selection bias. Generalisability of our study results especially to eastern regions of Turkey is limited. Another limitation is that the FPs and nurses participating in the study could not be selected from the same FHC. Therefore, when comparing FPs and nurses, the characteristics of the FHC and/or the region where the FHC was located could not be controlled. Finally, the results of our study may not be solely dependent on factors related to the pandemic, as we do not have data on the pre-pandemic burnout levels of our participants.

The findings of the study indicate that FPs and nurses are affected by burnout in different ways under the conditions of the COVID-19 pandemic, due to their gender, social status, living and working conditions. In addition to macro policy changes to address the current problems of health workers, interventions such as adequate and free PPE supply, providing the necessary and timely support for the organization of services and strengthen the PHC workers in emergency risk communication techniques should be planned for the next emergency.
